# Central venous catheter-related infection in a prospective and observational study of 2,595 catheters

**DOI:** 10.1186/cc3824

**Published:** 2005-09-28

**Authors:** Leonardo Lorente, Christophe Henry, María M Martín, Alejandro Jiménez, María L Mora

**Affiliations:** 1 Staff physician, Department of Intensive Care, Hospital Universitario de Canarias, La Laguna, Santa Cruz de Tenerife, Spain; 2 Methodological consultant, Research Unit, Hospital Universitario de Canarias, La Laguna, Santa Cruz de Tenerife, Spain

## Abstract

**Introduction:**

Central venous catheterization is commonly used in critically ill patients and may cause different complications, including infection. Although there are many studies about CVC-related infection, very few have analyzed it in detail. The objective of this study was to analyze the incidence of catheter-related local infection (CRLI) and catheter-related bloodstream infection (CRBSI) with central venous catheters (CVCs) according to different access sites.

**Methods:**

This is a prospective and observational study, conducted in a 24-bed medical surgical intensive care unit of a 650-bed university hospital. All consecutive patients admitted to the ICU during 3 years (1 May 2000 and 30 April 2003) were included.

**Results:**

The study included 2,018 patients. The number of CVCs and days of catheterization duration were: global, 2,595 and 18,999; subclavian, 917 and 8,239; jugular, 1,390 and 8,361; femoral, 288 and 2,399. CRLI incidence density was statistically higher for femoral than for jugular (15.83 versus 7.65, p < 0.001) and subclavian (15.83 versus 1.57, p < 0.001) accesses, and higher for jugular than for subclavian access (7.65 versus 1.57, p < 0.001). CRBSI incidence density was statistically higher for femoral than for jugular (8.34 versus 2.99, p = 0.002) and subclavian (8.34 versus 0.97, p < 0.001) accesses, and higher for jugular than for subclavian access (2.99 versus 0.97, p = 0.005).

**Conclusion:**

Our results suggest that the order for punction, to minimize the CVC-related infection risk, should be subclavian (first order), jugular (second order) and femoral vein (third order).

## Introduction

Central venous catheters (CVCs) are commonly used in critically ill patients for the administration of fluids, medications, blood products and parenteral nutrition, for the insertion of a transvenous pacing electrode and to monitor hemodynamic status. The use of catheters is habitual in critically ill patients; in the EPIC study, 78% of critically ill patients had some form of CVC inserted [1].

Central venous catheterization may cause different complications, including infection, haemorrhage and thrombosis. Interest in catheter-related infection lies in the mortality [2-5] and the costs [6-9] it represents.

In a previous study [10], our team analyzed catheter-related local infection (CRLI) and catheter-related bloodstream infection (CRBSI) resulting from the use of CVCs; these were reported for each site of CVC placement. The incidence density of CRLI in femoral or jugular sites was significantly higher than in the subclavian site; apart from this, there were no other significant differences between the use of CVCs at different sites. In the study presented here, we have increased the number of CVCs due to the probability of finding another significant difference.

Although there are many studies about CVC-related infection [11-31], we have found only two studies that have analyzed it in detail [11,12], but the number of CVCs used, 300 and 499 respectively, were lower than the 2,595 in our study.

The objective of this study was to analyze the incidence of CRLI and CRBSI at each central venous site.

## Materials and methods

A 3-year prospective study was performed that included all patients admitted to the 24-bed intensive care unit (ICU) of the Hospital Universitario de Canarias (Tenerife), between 1 May 2000 and 30 April 2003. The study was approved by the institutional review board.

The catheters used were not antimicrobial-coated, but were radiopaque polyurethane catheters (Arrow, Reading, PA, USA). The placement and maintenance of catheters were performed according to the following protocol. The catheters were inserted by physicians with the following sterile-barrier precautions: use of large sterile drapes around the insertion site, surgical antiseptic hand wash, and sterile gown, gloves, mask and cap. The skin insertion site was first disinfected with 10% povidone-iodine and anesthetized with 2% mepivacaine. The catheters were percutaneously inserted using the Seldinger technique and were fixed to the skin with 2-0 silk suture. After the line insertion, the area surrounding the catheter was cleaned with a sterile gauze soaked with povidone-iodine and a dry sterile gauze occlusive dressing covered the site. No topical antimicrobial ointment was applied to insertion sites.

The percutaneous entry sites were examined for the presence of local inflammation and purulence, and were cared for in the same manner daily by the ICU nurse assigned to the patient. Catheter dressings were changed every 24 h, or sooner at the discretion of the nurse caring for the patient if the dressing was contaminated. The connecting lines were changed every 48 h and disposable traducer components were replaced every 96 h.

Also, the percutaneous entry sites were examined daily by the ICU nurse assigned to the patient to avoid accidental catheter removals [32] in order to minimize infection risk associated with the reinsertion of the catheter.

The decision to remove the catheter was made by the patient's physician. Catheters were removed when they were no longer needed or if a systemic or local complication occurred. CVCs were routinely replaced every 14 days. We routinely used the guidewire technique to replace catheters, but in patients suspected of having a catheter-related infection the insertion site for the new catheter was changed. All catheter tips removed were routinely cultured. The catheters were removed using a sterile technique by an ICU nurse. The distal 5 cm segment of the catheters was cut with sterile scissors, placed in a sterile transport tube and cultured using the semi-quantitative method described by Maki *et al*. [33].

The following data were collected: age, sex, diagnosis, APACHE-II score, ICU admission and discharge dates, catheter access, catheter insertion and removal dates, cause of catheter removal, development of CRLI and CRBSI. The following three groups of CVCs were studied: femoral, jugular and subclavian.

Catheter-related infection was defined according to catheter tip colonization, CRLI or CRBSI. Catheter tip colonization was the significant growth of a microorganism (>15 colony-forming units) from the catheter tip. CRLI was any sign of local infection (induration, erythema, heat, pain, purulent drainage) and catheter tip colonization. CRBSI was a positive blood culture obtained from a peripheral vein, and signs of systemic infection (fever, chills, and/or hypotension), with no apparent source of bacteremia except the catheter, and catheter tip colonization with the same organism.

Statistical analysis was performed with SPSS 11.0 (SPSS Inc., Chicago, IL, USA) and LogXact 4.1 (Cyrus Mehta and Nitin Patel, Cambridge, MA, USA). Continuous variables are reported as means and standard deviation, and categoric variables as percentages. The CRLI and CRBSI rates are reported as: the percentage of catheters that developed CRLI; the number of CRLIs per 1,000 catheter-days; the percentage of catheters that developed CRBSI; the number of CRBSIs per 1,000 catheter-days. Comparison of the densities of incidence per 1,000 catheter-days, of CRLI and CRBSI, and between the different accesses were done using Poisson Regression and analyses were corrected for multiple testing with a Bonferroni correction. According to Bonferroni's adjustment, a p < 0.017 was considered statistically significant.

## Results

During the study period, 2,018 patients were admitted, of whom 1,243 (61.60%) were males. Their mean age was 56.85 ± 19.52 years; their mean APACHE II score was 13.81 ± 5.97; their mean length of ICU stay was 8.86 ± 13.18 days; and 262 (12.98%) patients died. Admission diagnoses were: 907 (44.95%) heart surgery; 278 (13.78%) trauma; 257 (12.71%) neurologic; 234 (11.60%) cardiologic; 199 (9.86%) respiratory; 91 (4.51%) digestive; and 52 (2.58%) intoxication.

The number of CVCs and days of catheterization duration were: global, 2,595 and 18,999; subclavian, 917 and 8,239; jugular, 1,390 and 8,361; femoral, 288 and 2,399. The incidence densities of CRLI and CRBSI were 6.05 and 2.79 per 1,000 catheter-days, respectively (Table 1).

As noted in Table 2, the CRLI incidence density was statistically higher for femoral than for jugular (15.83 versus 7.65, p < 0.001) and subclavian (15.83 versus 1.57, p < 0.001) accesses, and higher for jugular than for subclavian access (7.65 verus 1.57, p < 0.001).

Table 3 shows that the CRBSI incidence density was statistically higher for femoral than for jugular (8.34 versus 2.99, p = 0.002) and subclavian (8.34 versus 0.97, p < 0.001) access, and higher for jugular than for subclavian access (2.99 versus 0.97, p = 0.005).

A total of 53 microorganisms were responsible for the 53 CRBSIs, of which 38 (71.70%) were Gram-positive bacteria, 12 (22.64%) were Gram-negative bacteria and 3 (5.66%) were yeasts. Isolated from the 53 microorganisms were: 23 (43.39%) coagulase-negative staphylococci; 9 (16.98%) *Staphylococcus aureus*; 5 (9.43%) *Enterococcus faecalis*; 1 (1.89%) *Bacillus *spp.; 8 (15.09%) *Escherichia coli*; 2 (3.77%) *Enterobacter cloacae*; 2 (3.77%) *Pseudomonas aeruginosa*; and 3 (5.66%) *Candida albicans*.

## Discussion

The literature contains two studies that have analyzed catheter-related infection in detail [11,12], but the number of CVCs used in these (300 and 499 respectively) were lower than in our study (2,595 CVCs).

In some studies, 6% to 15% of CVCs developed CRLI [12-14]. The percentage of CVCs that developed CRLI in our study was somewhat lower (4.43%), probably because of our CRLI definition, which was more restrictive and required the presence of catheter-tip colonization.

We have found one study that reported a CRLI incidence density of 1.47 infections/1,000 catheter-days [11]; our CRLI incidence density was higher (6.05/1,000 days), probably because our definition was less restrictive and included only the presence of purulent drainage.

According to the literature, 1% to 13% of CVCs develop CRBSI [11-26] and the incidence density of CRBSI ranges from 2 to 4.5/1,000 catheter-days [27]. Our rates were near to this lower limit (2.04% CVC developed CRBSI and the CRBSI incidence density was 2.79/1,000 catheter-days).

Which venous catheterization site is associated with the highest risk of infection remains controversial. We have not found any study that looks at CRLI incidence with respect to different CVC accesses. Several studies have analyzed the catheter tip colonization (CTC) incidence according to different CVC accesses; in some studies, higher incidence occurred with femoral access [11,15,28,29]; in some it was higher with jugular access [12,19,20]; and others compared only jugular versus subclavian access, finding a higher incidence in the former [16,23,30]. Two studies that analyzed CRBSI incidence with respect to different CVC accesses found a higher incidence with femoral access [11,15].

In our study, femoral venous access was associated with a significantly higher incidence of CRLI and CRBSI than jugular and subclavian access; and jugular access was associated with a significantly higher incidence of CRLI and CRBSI than subclavian access.

Femoral vein access shows a higher incidence of CRLI and CRBSI than the other sites, probably because of the higher density of local skin flora in the groin area [29].

The higher incidence of CRLI and CRBSI with jugular access compared to subclavian access is probably due to three factors favoring skin colonization: the proximity of the insertion site to the mouth and the oropharyngeal secretion; the higher density of local skin flora due to the higher local skin temperature; and the difficulties in maintaining occlusive dressings [13,19,20].

Although the CDC guidelines of 1996 [34] and 2002 [35] recommend against routinely replacing CVCs to prevent catheter-related infections, we routinely changed CVCs every 14 days for two reasons: first, in several studies, central venous catheterization longer than 5 to 7 days was associated with a higher risk of catheter-related infection [12,13,20,31]; and second, in other studies, CVCs were routinely changed every 7 or 10 days [17,30].

All catheters analyzed were inserted under maximal sterile barrier precautions because there is evidence that this method reduces the risk of catheter infection [36]. Catheters placed under emergency situations, during which optimal aseptic conditions cannot always be fully respected, have been significantly associated with higher risk of catheter-related infection [11,19]. Because of this, catheters placed under emergency situations were replaced as soon as possible and, to avoid a major bias in the catheter-related infection incidence between the different access sites, were eliminated from the statistical analysis, as in another studies [13,15]. The CDC guidelines of 1996 [34] made no recommendation for the removal of CVCs and arterial catheters (ACs) inserted under emergency conditions (it was considered an unresolved issue), but for peripheral venous catheters they recommended that they should be removed and a new catheter inserted at a different site within 24 hours. The current CDC guidelines of 2002 [35] recommend that when adherence to aseptic technique cannot be ensured (i.e., when catheters are inserted during a medical emergency), all catheters should be replaced as soon as possible and after no longer than 48 hours.

In our series (since 1 May 2000 until 30 April 2003), the gauze dressings were changed every 24 hours because the CDC guidelines of 1996 did not include any recommendation regarding the frequency of routine replacements of dressing (it was considered an unresolved issue), although the CDC guidelines of 2002 recommend that gauze dressings be replaced every 2 days (category IB) because frequent dressing changes have been shown to increase the risk of catheter infection [37,38].

Our study includes three limitations. First, different insertion sites were not randomly assigned. No randomized trials, however, have compared infection rates for CVCs placed in the three different sites. Only in the study of Merrer *et al*. [15] were patients randomly assigned to undergo CVC at the femoral or subclavian site. Second, the absence of a multivariate analysis to control for possible confounders. And third is the CRLI definition we have used. Our definition of CRLI included both any sign of local infection and a positive semi-quantitative culture of the catheter tip. This definition is one of the possible criteria for venous infection according to the 1988 CDC guidelines [39]. The CDC guidelines of 1996 [34] and 2002 [35], however, did not require a positive culture of the insertion site as part of the CRLI definition, but did distinguish the following aspects of CRLI: exit site infection, pocket infection and tunnel infection.

## Conclusion

In the CDC guidelines of 1996 and in the latest guidelines of 2002, CVC insertion at the subclavian site is recommended rather than at the femoral or jugular sites to minimize infection risk. Our results suggest that the order for punction, to minimize CVC-related infection risk, should be subclavian (first order), jugular (second order) and femoral (third order).

## Key messages

• 	To minimize catheter-related infection, it is necessary to monitor its incidence and to implement preventive measures.

• 	We found that the femoral venous access was associated with a significantly higher incidence of CRLI and CRBSI than the jugular and subclavian venous accesses.

• 	We found that the jugular venous access was associated with a significantly higher incidence of CRLI and CRBSI than the subclavian access.

• 	Our results suggest that the order for punction, to minimize the CVC-related infection risk, should be subclavian (first order), jugular (second order) and femoral (third order).

## Abbreviations

APACHE = Acute Phisiology and Chronic Health Evaluation; CRBSI = catheter-related bloodstream infection; CRLI = catheter-related local infection; CVC = central venous catheter; ICU = Intensive care unit.

## Competing interests

The authors declare that they have no competing interests.

## Authors' contributions

LL conceived and designed the study, and was involved with acquisition of data, analysis, and interpretation of data. CH was involved with acquisition of data and drafted the manuscript. MMM was involved with acquisition of data and drafted the manuscript. AJ was involved with analysis and interpretation of data. MLM conceived and designed the study and was involved with the interpretation of data. All authors gave final approval of the version to be published.

## Figures and Tables

**Table 1:** Catheter-related local infections and catheter-related bloodstream infections with central venous catheters inserted at various sites

Site	Number of CVCs	Days of CVC	Number of CRLIs	ID of CRLIs	% CVC with CRLI	Number of CRBSIs	ID of CRBSIs	% CVC with CRBSIs
Subclavian	917	8,239	13	1.57	1.42%	8	0.97	0.87%
Jugular	1,390	8,361	64	7.65	4.60%	25	2.99	1.80%
Femoral	288	2,399	38	15.83	13.19%	20	8.34	6.94%
								
Total	2,595	18,999	115	6.05	4.43%	53	2.79	2.04%

CRBSI, catheter-related bloodstream infection; CRLI, catheter-related local infection; CVC, central venous catheter; ID, incidence density defined as number of infections per 1,000 catheter-days.

**Table 2:** Comparison of catheter-related local infection incidence densities between different central venous sites

	ID	OR (95% CI)	P-values
Femoral versus jugular	15.83 vs 7.65	2.1 (1.35–3.14)	<0.001
Femoral versus subclavian	15.83 vs 1.57	3.2 (2.29–4.53)	<0.001
Jugular versus subclavian	7.65 vs 1.57	4.8 (2.64–9.60)	<0.001

CI, confidence interval; ID, incidence density defined as number of infections per 1,000 catheter-days. OR, odds ratio.

**Table 3:** Comparisons of catheter-related bloodstream infection incidence densities between different central venous sites

	ID	OR (95% CI)	P-values
Femoral versus jugular	8.34 vs 2.99	2.8 (1.46–5.22)	0.002
Femoral versus subclavian	8.34 vs 0.97	2.9 (1.90–4.75)	<0.001
Jugular versus subclavian	2.99 vs 0.97	3.1 (1.34–7.90)	0.005

CI, confidence interval; ID, incidence density defined as number of infections per 1,000 catheter-days. OR, odds ratio.
